# Exploring stroke risk and prevention in China: insights from an outlier

**DOI:** 10.18632/aging.203096

**Published:** 2021-06-04

**Authors:** Xinrou Lin, Hongxuan Wang, Xiaoming Rong, Ruxun Huang, Ying Peng

**Affiliations:** 1 Department of Neurology, Sun Yat-Sen Memorial Hospital, Sun Yat-Sen University, Guangzhou, China; 2 Department of Neurology, The First Affiliated Hospital, Sun Yat-Sen University, Guangzhou, China; 3 Guangdong Provincial Key Laboratory of Malignant Tumor Epigenetics and Gene Regulation, Sun Yat-Sen Memorial Hospital, Sun Yat-Sen University, Guangzhou, China

**Keywords:** stroke, hypertension, atrial fibrillation, antiplatelet, cancer

## Abstract

In contrast to the declining trend in most regions worldwide, the incidence of stroke is increasing in China and is leading to an alarming burden for the national healthcare system. In this review, we have generated new insights from this outlier, and we aim to provide new information that will help decrease the global stroke burden, especially in China and other regions sharing similar problems with China. First of all, several unsolved aspects fundamentally accounting for this discrepancy were promising, including the serious situation of hypertension management, underdiagnosis of atrial fibrillation and underuse of anticoagulants, and unhealthy lifestyles (e.g., heavy smoking). In addition, efforts for further alleviating the incidence of stroke were recommended in certain fields, including targeted antiplatelet regimes and protections from cold wave-related stroke. Furthermore, advanced knowledge about cancer-related strokes, recurrent strokes and the status preceding stroke onset that we called stroke-prone status herein, is required to properly mitigate patient stroke risk, and to provide improved outcomes for patients after a stroke has occurred.

## INTRODUCTION

**Figure 1:**
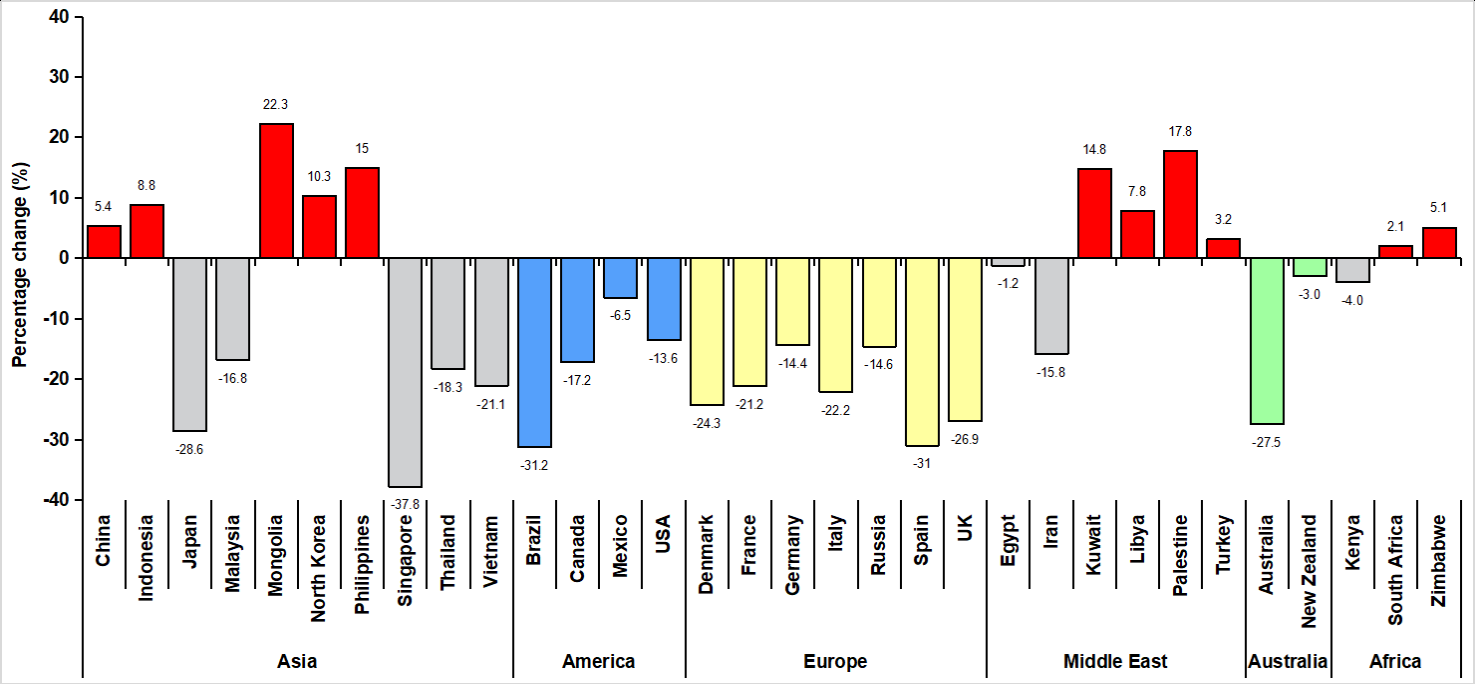
Percentage change in age-standardized incidence rates of overall stroke in different countries, 1990-2016, extracted from a systematic analysis for the Global Burden of Disease Study 2016 [1].

Stroke brings notable health burden all around the world. It is the second leading cause of death and disability-adjusted life-years (DALYs) globally (5.5 million deaths, 116.4 million DALYs in 2016), but the first leading cause of death and DALYs in China (1.79 million deaths, 38.6 million DALYs in 2016) [1–3]. With striking improvement of strategies in prevention and specialist stroke care, China has experienced a transition in the epidemiology of stroke [4]. Its downtrend of stroke mortality is on par with the global tendency [1, 5]. However, the incidence rate has increased over the years and continues to increase, which are on the contrary with the decline trend in most regions worldwide [1, 5, 6]. (Figure 1) With over 5.5 million new cases in 2016, China had the highest age-standardized incidence of stroke [1]. Moreover, the incidence rate increases by 8.3% annually, which leads to a dramatic growth in stroke prevalence and substantial burden for both patients and the health system [4, 6, 7]. The upward tendency of stroke incidence in China has become a severe health problem and urges the optimization of managements.

In this review, we adopt updated data to highlight several most potential aspects of stroke risks and preventions in China from a comprehensive and comparative perspective. In comparison to those in western countries, the current situation of hypertension, underdiagnosis of atrial fibrillation and unhealthy lifestyles in China are serious; the use of antihypertensive therapy and anticoagulant therapy are insufficient in China. However, these approaches represent promising opportunities to decrease stroke incidence in China, if progress towards their wide-scale implementation are achieved. Besides, targeted antiplatelet regimes for different subpopulations and specific protections from cold waves will mitigate the morbidity of stroke. In addition, advanced knowledge for pathogenesis, identification and preventions of cancer-related stroke and recurrent stroke are required since their occurrence appears to be increasing in Chinese populations. Moreover, we introduce hypertension as a cause of stroke and a tentative concept of so-called stroke-prone status. Overall, our review is designed to provide guidance, insights and new information to patients, physicians, policymakers, and researchers aiming to decrease the increasing stroke incidence in China and other regions sharing similar situations including Southeast Asia and sub-Saharan Africa [1].

## Serious situation of hypertension

Hypertension is viewed as the greatest modifiable risk factor for stroke generally [2, 4, 6, 8]. However, on the basis of animal experiments and epidemiological data displaying a strong association between hypertension and stroke, we conceive that hypertension can be viewed as more of a single cause of stroke than a risk factor. Firstly, high blood pressure was proven to be able to cause stroke pathologically in animal models via cerebral vascular remodeling, increased permeability, sclerosis of cerebral vessels and so on [9–12]. Then, large-scare surveys exhibited that over 84% of stroke patients had a history of hypertension when they were enrolled, and meanwhile, 71% of hypertensive patients in the Chinese population died of cerebral vascular accidents [8, 13, 14]. Moreover, the stronger association between hypertension and stroke in Chinese populations than in Whites corresponded to the severer situation of stroke [15–17]. Hypertension is associated with higher hazards in intracerebral hemorrhage (ICH), and consistently there were two fold proportion of ICH patients in China [15, 16]. The mean blood pressure was higher in China (130.0/76.4 mm Hg) than in America (125.1/72.5mm Hg) [18]. The severe blood pressure levels (≥160/100 mm Hg) were nearly 2.5 fold more common in China than in America [18]. When systolic blood pressure (SBP) elevated each 10mmHg, the risk of ischemic stroke (IS) increased about 30% and the risk of ICH increased about 60% in China [19], whereas the increase hazard of overall stroke was only about 20% in Whites [20]. Besides, not just in China, stronger association between hypertension and stroke was also revealed in regions sharing with elevated stroke incidence (e.g., Southeast Asia and Africa), on the evidence that their population-attributable risk (PAR) and odds ratio (OR) of hypertension for stroke exceeded that in western countries [16].

**Figure 2:**
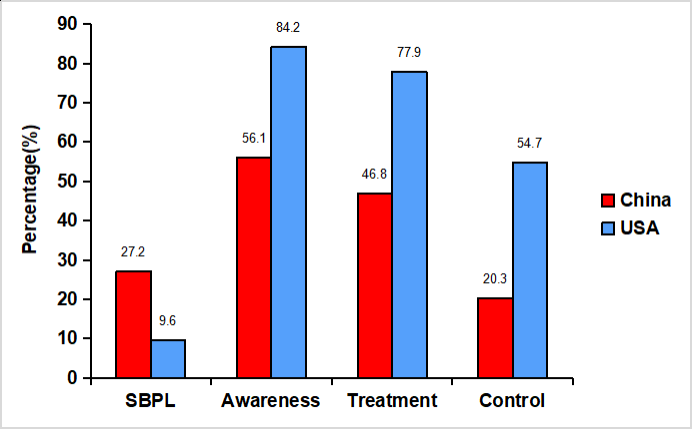
**Comparisons among hypertensive patients between China and the USA during 2011 to 2012.** SBPL: severe blood pressure levels (≥160/100 mm Hg). Percentage (%) in columns means: 1) SBPL: blood pressure of ≥160/100 mm Hg among hypertensive people (prevalence of severe hypertension among all people/prevalence of hypertension among all people). 2) Awareness: a self-reported physician diagnosis of hypertension or self-reported use of antihypertensive medication, among hypertensive people. 3) Treatment: self-reported use of antihypertensive medication, among hypertensive people. 4) Control: a mean systolic blood pressure of ≤140 mm Hg, and a mean diastolic blood pressure of ≤90 mm Hg, among people with previously diagnosed hypertension. Adapted from a study using data from CHARLS (China Health and Retirement Longitudinal Study, n=12654) and the NHANES (US National Health and Nutrition Examination Survey, n=2607) from 2011 to 2012 [18].

Albeit the awareness, treatment and control rates of hypertension among Chinese patients has dramatically increased, they are lower than that in western countries [7, 21]. For example, compared with the United State, China had considerably lower rates of hypertension awareness, treatment and control [18, 22]. (Figure 2) The greatest backwardness was the poor control of hypertension that might largely result from more common use of monotherapy antihypertensive drug regimens. In comparison to the monotherapy, single-pill combinations and multiple-pill combinations of antihypertensive drugs increased 55% and 26% likelihoods of better control of blood pressure, respectively [23]. However, merely 21.9% of Chinese hypertensive patients took multiple antihypertensive drugs in 2014 to 2017, while the percentage was up to 47.7% among American in 2009 to 2010 [22, 23]. About 81.5% of hypertensive patients that were treated but not controlled were those who received only one antihypertensive agent [22]. And equally, the remarkable achievement of hypertension control in America is now attributed to increasing use of multi-drug therapy [23].

Overall, hypertension might play a dominant role in stroke occurrence as a single direct cause and the inadequate use of antihypertension increases stroke onset related to uncontrolled hypertension. Rigorous interventions including but not limited to improving availability of antihypertensive medications and increasing prescription of high-value medications are required [24].

## Underdiagnosis of atrial fibrillation and underuse of anticoagulants

Atrial fibrillation (AF) is associated with 5-fold risk for stroke and contributes to over 40% of stroke in patients older than 80 years [25, 26]. The excess risk of ischemic stroke due to AF was greater in China (OR 6.98, 95%CI 3.46- 14.1) than in Europe and America (OR 4.05, 95%CI 2.55-6.44) [16]. However, in the setting of increasing prevalence of AF internationally, the lowest prevalence of AF was estimated in China [16, 27, 28]. Compared with other risk factors, AF presented exceptionally lower prevalence in Chinese stroke patients than in white populations [15]. The prevalence of ischemic stroke with AF was about 10% while it was about 26~27% in Whites [29–32]. The gap lies more in the frequency of overlooked or underdiagnosed AF, rather than the low absolute number of patients [33]. This can be ascribed to the bulk of patients receiving 12-lead electrocardiography only. To enhance the identification and diagnosis is of significance since AF-related stroke can be prevented by initiating anticoagulation. Besides, a quarter of ischemic stroke is cryptogenic and a proportion of presumed cryptogenic transient ischemic attack (TIA) or stroke can be attributed to under-recognized atrial fibrillation [34–36]. And without recognized history of AF at discharge, stroke patients had 40% higher risk of stroke recurrence during one-year follow-up period [37]. Therefore, it is of necessity to intensify the detection of atrial fibrillation particularly transient paroxysmal and clinically silent AF. Advanced monitoring such as opportunistic or systematic screening, mobile cardiac outpatient telemetry and prolonged cardiac rhythm monitoring should be applied if appropriate [26, 33, 34, 38].

The use of oral anticoagulant therapy among new-onset AF patients in China was notably lower than that in other countries, and even failed to achieve the global average level [31, 39]. Even among stroke patients with non-valvular atrial fibrillation (NVAF), the utilization of anticoagulants was insufficient. Adjusted-dose warfarin and antiplatelet agents reduced stroke by 64% and 22%, respectively [40], but only 35.5% received warfarin but 62.7% received antiplatelet therapy among Chinese AF patients receiving antithrombotic therapy [41]. At discharge, merely 19.4% of stroke patients with NVAF in China received warfarin [32] whereas the proportion was 88% in America [42]. The underuse of warfarin mainly resulted from concern about higher bleeding risk in Asian population among doctors and patients [6, 32, 43]. Besides, owing to inadequate monitoring of INR and usage of diverse traditional Chinese herbals, about 55.2% of Chinese NVAF patients receiving warfarin did not reach the therapeutic range of INR [31]. New oral anticoagulants (NOAC), with superiority for stroke prevention, less side effects, fewer drug interactions and needlessness to surveil the INR, are better choices for Chinese patients [44]. However, only when NOAC are affordable will they be commonly used and their adherence be guaranteed. Overall, the underdiagnosis of AF combined with underuse of anticoagulants may cause an increase in stroke cases that could easily be prevented.

## Unhealthy lifestyles

As a risk factor for hypertension and stroke, high sodium consumption was a serious issue in China [45, 46]. The intake of 8 to 9 g/d in the south and 12 to 18 g/d in the north entirely surpassed World Health Organization recommendation of 5g/d limit [46]. Salt and soy sauce are core condiments in Chinese Cuisine and salted food remain common in daily lives of rural people.

Current smoking conferred a PAR of 15% for stroke in China [16]. Even though the smoking prevalence and second-hand smoke exposure had reduced together with increasing percentage of ex-smokers over the years [47], Chinese was still the largest ethnic group of current smokers and made up a third of global male smoking populations [48]. The consumption of cigarettes in China was more than the combination of the next 40 highest consuming countries from 1970 to 2015, and continued to increase [49–51]. Furthermore, second-hand smoke exposure due to tobacco sucked in China brings larger hazards than that chewed in India where the number of smokers ranked second worldwide [49]. Ultimately, it may be very difficult to control tobacco use in China for several reasons. Tobacco is considered the third most addictive drugs to human [52], and there is also a widespread culture of use in China, where tobacco serves as a popular gift in traditional festivals to enhance relationships and a mediator to entertain clients in social intercourses.

Alcohol, another one of the top ten addictive drugs [52], is another risk factor for stroke especially intracerebral hemorrhage [15, 16]. Although the prevalence of weekly drinking decreased from 2004-2008 to 2013-2014, the mean alcohol consumption, drinking frequency and heavy episodic drinking prevalence are increasing in China, particularly among the young generation [53]. In the past three decades, the alcohol per-capita consumption has reduced in America and Europe, while it has risen in China [54, 55]. Similar to smoking, drinking alcohol especially strong spirits (e.g. Baijiu) is also a symbol of celebration and capacity in Chinese traditional culture [53, 55].

Driving motorcycles and private cars are also more attractive than walking and cycling to the Chinese, and these activities can exacerbate physical inactivity and increase exposure to air pollution, which are risk factors for stroke [6, 45]. Completely exceeding other regions, China presented the highest PAR (59.9%) of physical inactivity for stroke [16]. In addition, the concentration of PM2.5 in China was the second highest among 79 countries when studies showed that PM2.5 precipitated the incidence of stroke [6, 56].

Traditional, but less-healthy, lifestyles have existed in China for a long time, and many of these cultural living patterns may be at odds with modern approaches to stroke prevention [45]. Unhealthy lifestyles are increasingly common in younger generations of Chinese, which partly explains the younger onset of stroke in China than in other regions [4, 6, 15]. It is time to launch a nationwide campaign against the unhealthy lifestyles since they increase the occurrence of stroke. Advertising their hazards for health to the public, seeking for alternatives like salts with potassium chloride or magnesium sulfate, strengthening the price and excise tax of tobacco and alcohol, and increasing the use of clean fuels like gas and electricity are feasible actions [55, 57, 58]. Indeed, there are numerous opportunities for reducing stroke risk and improving health outcomes in China, however it may be difficult to change the rigid lifestyle patterns of the traditional Chinese culture which is contributing to the disease.

## Antiplatelet therapy to be targeted

Valid antiplatelet drugs for primary prevention of stroke remain unclear. Taking aspirin daily was not beneficial for primary stroke prevention, but instead increased the risk of hemorrhagic stroke and gastrointestinal bleeding [59, 60]. Nevertheless, antiplatelet therapy plays a dominant role for the prevention of stroke recurrence and complications.

Aspirin, as the class 1 recommendation, was the first antiplatelet agent used for the secondary prevention of TIA and ischemic stroke. It decreases long-term risk of recurrent stroke by 13% and risk of early recurrent stroke by 60% [61]. Clopidogrel, a commonly prescribed and used antiplatelet agents, has greater effectiveness in stroke prevention and less side effects than aspirin [62]. The genetic polymorphisms of CYP2C19 account for the variable efficacy of this prodrug [63]. CYP2C19 loss-of-function allele (*2 and *3) carriers are poor metabolizers with reduced enzyme activity and an increased risk of composite vascular events [64]. Disappointingly, the frequency of such poor metabolizers was up to 58.8% among Chinese, which was overwhelmingly higher than that in Caucasians and African Americans [64, 65]. Dual antiplatelet therapies such as aspirin plus clopidogrel, aspirin plus dipyridamole, aspirin plus ticagrelor were superior to monotherapies in the therapeutic efficacy, but the frequency of bleeding events and discontinuation from trial medications increased, especially in Asian populations [66–69]. Hence, aspirin monotherapy is still the footstone for the prevention after stroke.

Although the effectiveness of antiplatelet regimes for stroke prevention have been demonstrated in large randomized controlled trials (RCT), it is not always applicable for every ethnic group that has divergent genetic profile. Although the utilization of antiplatelet agents in Chinese patients was always at a high level in different studies, this does not mean that the treatment functions consistently or side effects can be offset by efficacy [6]. Only by choosing targeted antiplatelet therapies for different subpopulations will benefits ensue maximally, especially when various antiplatelet regimes are emerging. Population-based investigations about options of different antiplatelet therapies pertaining to specific ethnic groups are required.

## Neglect of cold wave-related stroke

Studies revealed that cold waves can increase the risk of stroke onset via vasoconstriction and blood viscosity [70–72]. Experiments established that artificial cold exposure (ACE) induced stroke in Reno vascular hypertensive rats [71]. Epidemiologic data described that a large fluctuation of temperature had a strong association with cerebrovascular risk and increased hospitalization of stroke [72]. Compared with heat exposure and low ambient temperature, the cold wave that is defined as a rapid drop of temperature, poses greater burden on stroke [70, 73]. The risk of ischemic stroke increases by 11% for each 2.9° C temperature decrease over 24h [74], and the risk of intracerebral hemorrhage increases by 16.5% for each 5° C temperature decrease among people over 65 years old [72].

Cold wave events are predicted to be more frequent as a result of anthropogenic global warming and the super EI Nino event in East Asia in 2016 [75]. On the other hand, the human body temperature tends to decrease [76]. This gap between the increasing frequency of cold waves and decreasing human body temperature may lead to increasing occurrence of stroke due to weaker defense against weather change. Hence, the previously underemphasized environmental factor of cold wave on stroke incidence grows to be more important. Although cold wave events are unexpected and unpredictable, the lag effect of stroke after cold wave exposure creates opportunity for stroke preventions to minimize the hazards during post-disaster periods [70, 72].

Stroke burden attributed to cold temperature was greater in the southern China (e.g. Guangzhou) than in northern areas (e.g. Beijing) due to a lack of adaptability to cold temperature in subtropical population [70, 73, 75, 77, 78]. There was no ongoing pertinent protection plans towards the rapid temperature decline at a regional level in China. Weather forecasts emphasizing big changes of ambient temperature, short-term use of convenient heaters (e.g. blankets and hand warmers) to vulnerable subpopulations in southern areas and convenient heating services with clean fuels in northern areas are cost-effective measures [70, 75, 77]. Moreover, potential biomarkers like Syt1 and Idh3a could be considered to be applied in clinical practice for early identification of cold wave-related stroke among inpatients during cold waves, and prophylactic drugs like batroxobin for treatment of cold wave-related stroke [71, 79].

## Prospect of recurrent stroke

Recurrent stroke accounts for over 20% of patients admitted to hospital with an acute stroke event [80, 81]. With worse outcomes in disability and fatality, recurrent stroke was at a frequent level and became more frequent than before in China while decline trends were showed in Sweden, Italy, and Scotland [82, 83]. For example, incidence of recurrent stroke in 2006-2012 was three times higher than that in 1992-1998 in rural China [83]. Nevertheless, updating data from large-scale observational studies that represent the whole population of China is needed.

The combination of the use of aspirin, smoking cessation, antihypertensive drugs, and statins reduced the risk of recurrent ischemic events by 75% [45]. In contrast, a recent international RCT(INSPiRE-TMS) yielded a partly divergent finding. Regardless of improved achievement of recommended secondary prevention targets in the long term, including blood pressure, LDL, physical activity and smoking cessation, a support programme for enhanced secondary prevention did not bring a significant reduction in major vascular events [81]. The conflict between studies otherwise reflects that towards risk factors, primary preventions for stroke count more than secondary preventions. Therefore, it makes sense that neglect of recent minor stroke or TIA, under-recognized of atrial fibrillation, inadequate antithrombotic therapies, and failure of long-term adherence to treatments precipitates the severity of recurrent stroke in China. Further studies are required to ascertain whether achieving the secondary prevention targets is beneficial to prevent recurrent stroke, and explore whether the targets should be more aggressive for those with a high risk of recurrence, whether there are more advanced interventions and whether it is necessary to positively detect stroke recurrence.

## More frequency of cancer-related stroke

Cancer patients have twofold higher risk of stroke than the general population through special mechanisms: cancer embolus, leukostasis in intracranial vessels, compression of tumor, hypercoagulability, non-bacterial thrombotic endocarditis and special therapies including radiation and cytotoxic drugs [84, 85]. Stroke could occur in almost every cancer type, among which brain tumor and lymphoma (particularly children) are the two commonest cancers at <40 years of age, while prostate, breast, and colorectal tumor are the commonest cancers at >40 [85]. The average time interval from cancer diagnosis to stroke onset was 39.7 ± 60.9 months and the risk went up with longer follow-up time in cohort studies [85, 86].

**Figure 3:**
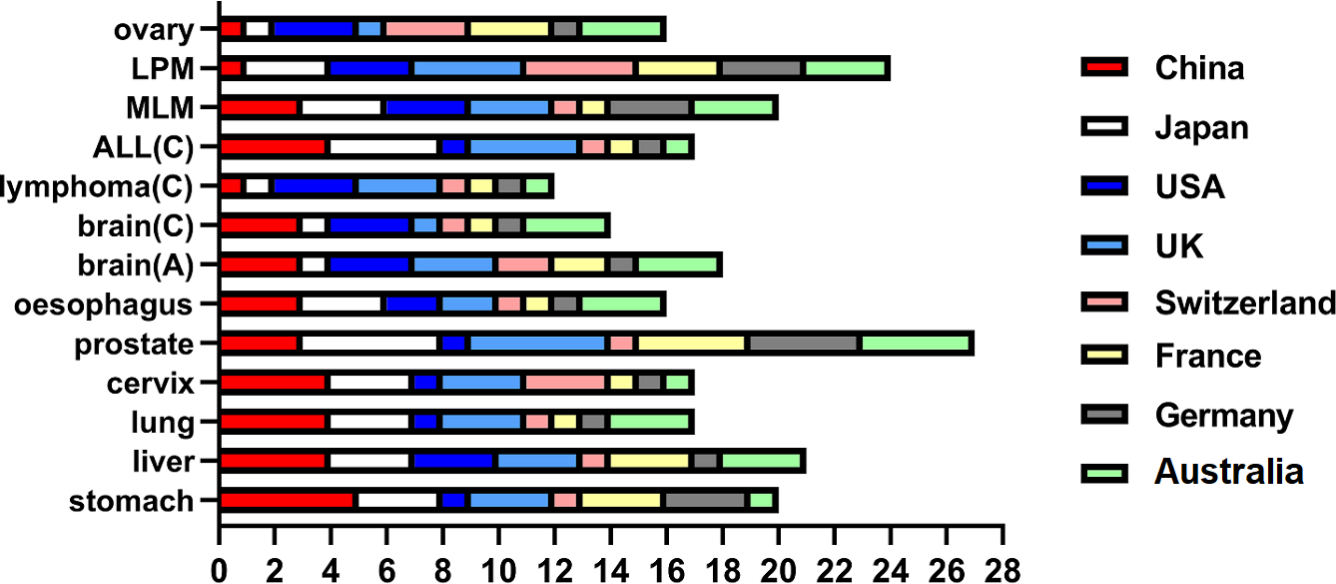
**Age-standardized 5-year survival increases of cancers in different countries, 2000-2014.** ALL: acute lymphoblastic leukemia; LPM: lymphoidmalignancies; MLM: myeloidmalignancies; (C): children; (A): adult. The percentage of survival increases are classified as: 1 score: no increase; 2 score: 0-5%; 3 score: 5-10%; 4 score: 10-20%; 5 score: >20%. Data are extracted from the third cycle of the CONCORD programme (CONCORD-3) that established global surveillance of trends in cancer survival 2000-2014 [88].

With advances in the care quality and health expenditures of cancers, the age-standardized 5-year survival for all cancers combined in China had significantly increased over the years, particularly in rural areas [87]. Even though the survival rate in China was not at a very high level at present, the 5-years survival increase of most cancers and the absolute number of cancer survivors were higher than those in other countries [88]. (Figure 3) It thus increases the number of potential cancer-related stroke patients, especially when the cancer tends to be a chronic disease that does not directly cause the patient’s death. Further population-based investigations about the epidemiology of cancer-related stroke conducted in China are needed.

The sudden onset of stroke among cancer patients may delay the scheduled surgery or chemotherapy, and thus adds severity and complexity to treatments of cancers, or even cause death and make the endeavor against tumors for saving lives appear to have been in vain. Specific identifications, managements and preventions for cancer-related stroke are limited for clinicians, researchers and patients currently, but they are expected as the prevalence and survival of cancer continue to increase.

## Concept of stroke-prone status

**Figure 4:**
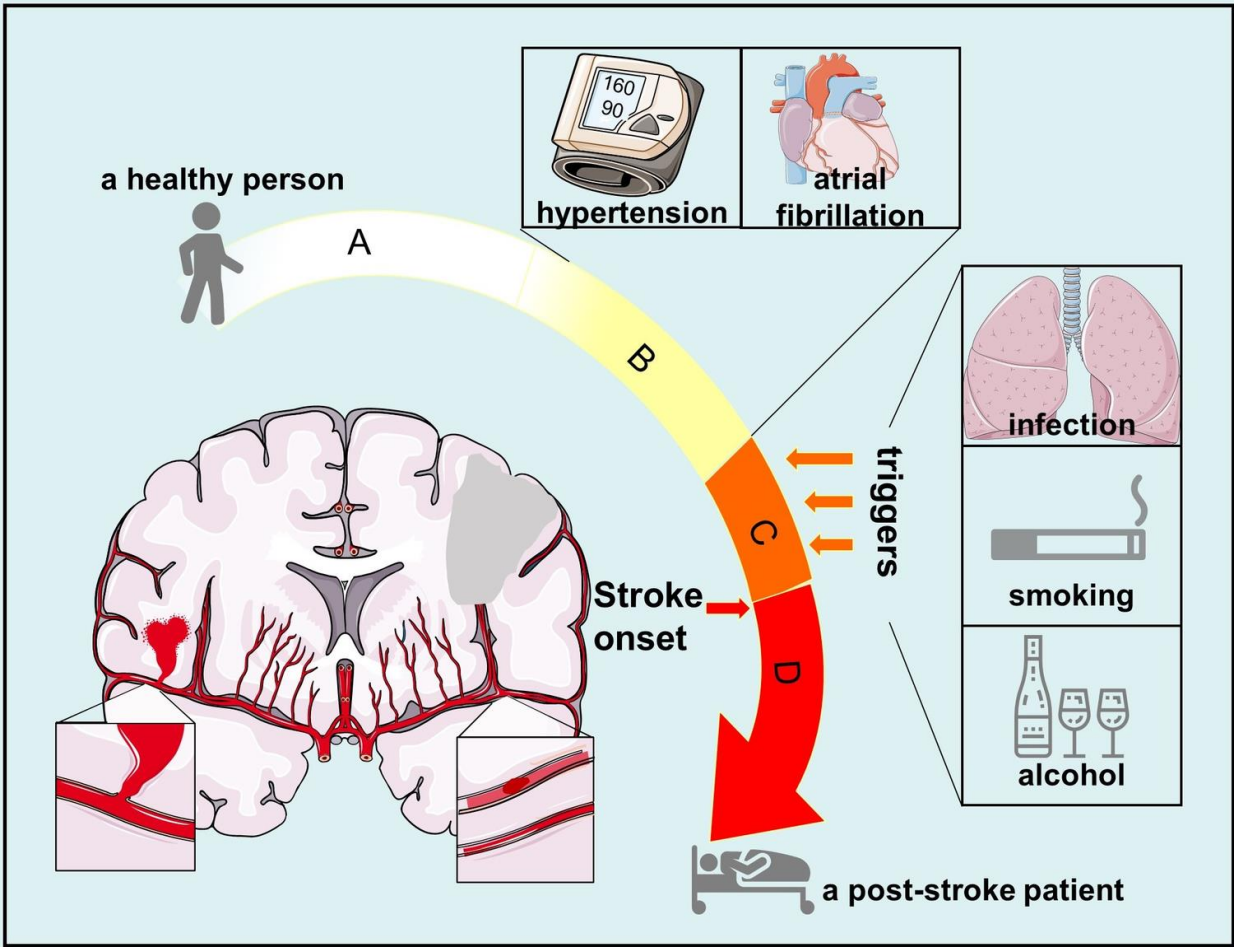
**Process from normal status to post-stroke status.** (**A**) normal status when a person is healthy and has no chronic disease; (**B**) status when a person suffers from diseases that are risk factors of stroke, including hypertension, atrial fibrillation and so on; (**C**) stroke-prone status when outside attacks such as infection and alcohol can trigger stroke onset in a person with risk factors of stroke; (**D**) post-stroke status when a person has suffered from stroke onset.

Stroke triggered by several conditions, encompassing acute infections, temperature decline and intensive unhealthy behaviors were elucidated in studies [89–92]. Solly Sebastian et al. revealed that the association between infection and stroke increased as their time interval shortened; A portion of patients had heavy alcohol intake within 24 hours preceding stroke onset; Moderate to extreme physical exertion tripled the risk of subarachnoid hemorrhage in the subsequent two hours [90–92]. However, exposure to similar triggers does not necessarily lead to similar outcome of stroke events, such as among the healthy population and certain patients with similar underlying diseases like hypertension or atrial fibrillation. Therefore, we presume that prior to the acute episode of stroke, there is a period when the internal environmental hemostasis is susceptible to outside attacks and is approaching the threshold of stroke onset. Then, when the host is exposed to triggers, critical inner pathophysiological changes provoke progressively, leading to an arrival of threshold and occurrence of subsequent stroke, which makes things change from quantity to quality. Herein, we call this period the stroke-prone status. It is a status when accumulation of inner changes is sufficient for triggers and is approaching the threshold of stroke onset (Figure 4).

If individuals that are at the stroke-prone status suffer from triggers, subsequent stroke events are possible to occur. If individuals are at the stroke-prone status though they do not suffer from any triggers, internal environmental hemostasis changes at a low pace and stroke will not occur until the inner changes reach the threshold. For example, some patients suffer from stroke when they are sleeping. But if individuals are not at the stroke-prone status though they suffer from trigger conditions, stroke is less likely to occur, including the healthy population and patients with risk factors but not suffering from stroke in their whole lives. Additionally, animal experiments displayed that stroke prone Reno vascular hypertensive rats did not have stroke onsets until the fourth times of natural coldness [93]. This further suggested that there exists stroke-prone status before stroke onset and a threshold for stroke onset.

Regarding hypertension, there are periods of blood pressure higher than normal levels but not enough for hypertension diagnosis. Regarding diabetes, there is prediabetes called impaired glucose tolerance when blood sugar is higher than it should be but not enough to diagnose diabetes. Regarding acute myocardial infarction, there are conditions of coronary artery stenosis and angina lasting for some time. However, there are no periods identified before the acute onset of stroke, but it is essential for stroke prevention especially when stroke is still a heavy health burden with alarming incidence and prevalence. The identification of stroke-prone status can be a combination of clinical symptoms like fatigue, dizziness, malaise, experimental examinations like viscosity of blood, platelet reactivity, stenosis of cerebral vessels and thrombus in left atrium, and history of underlying diseases like duration of hypertension and atherosclerosis burden. Further research about details of stroke-prone status and threshold of stroke onset are required for exploration. If it is able to assess the stroke-prone status quantitatively and then appoint specific preventions at this period, large numbers of stroke incidence can be prevented.

## Increase of the aging population

As the country with the highest population, China is facing with challenges of ascending rate of aging population. The Old Dependency Ratio in China (percent of 65-years and older to working ages) had increased from 9.9% in 2000 to 16.8% in 2018 [94], which was higher than the average ratio of the world (13.6% in 2018) [95]. This can be result from two of the three peak periods of population growth in 1950s to 1970s. A mass of babies born during those days are now entering their old age when stroke incidence is the highest among all age groups [8, 94].

In spite of China, aging has become a universal public problem in developed countries. Although aging is the irreversible risk factor of stroke, multiple modifiable health-related factors are concomitant in the elderly which should be paid more attention to. For example, the comorbidity with coronary heart disease, diabetes mellitus, dyslipidemia, mood or sleep disorders, physical inactivity and so on is common in the elderly. These factors all predispose elderly to higher risk of stroke. However, awareness and management of these aging-related comorbidities was less satisfying in China than those in developed countries [6, 16, 18, 45].

## CONCLUSIONS

The increasing stroke incidence in China in contrast to the global trend has brought alarming and ascending burden to the health system. In this review, we discuss several of the most prominent causes of stroke facing the Chinese, and we highlight several opportunities to reduce stroke incidence through public outreach and education. Firstly, several unsolved aspects showing stronger associations with stroke occurrence in China have marked deficiencies in comparison to those in western countries. Hypertension is serious issue in China, among which the greatest challenges is reduced control of hypertension compared to other developed nations, due to the less frequent use of multiple antihypertensive medications. The underdiagnosis of atrial fibrillation and underuse of anticoagulants are much more common. Unhealthy lifestyles as parts of traditional cultures for years are still severe health issues. Secondly, targeted antiplatelet therapies for different subpopulations as well as protections from cold wave and preventions from weather-related stroke will further allay the stroke occurrence. Thirdly, the frequency of recurrent stroke is high and is increasing but, secondary prevention strategies aimed to reduced recurrent stroke remain elusive and related clinical trials are sparse. Cancer-related stroke also appears to be increasing but, advanced interventions and related research are limited. Finally, we introduce two perspectives: high blood pressure viewed as a direct cause of stroke; and a tentative concept called stroke-prone status. If individuals with risk factors are at stroke-prone status, they are susceptible to triggers and approaching the subsequent stroke onset.

In order to alleviate the stroke incidence in China, education is of paramount significance to increase individuals’ awareness of health. It is important to emphasize healthy lifestyles, adherence to drug therapies and protections from environmental events to the public, especially among the young generation and patients in rural areas. Physicians should enhance the diagnosis and treatment of hypertension and atrial fibrillation, formulate individualized regimes of antithrombotic drugs and provide more supports to patients under high risk of stroke. Policymakers should enforce vigorous policies for tobacco and alcohol cessation, carry out regional strategies to protect citizens from climate changes and increase health care expenditures. Further research is required to focus on aspects with limited advances, comprising recurrent stroke, cancer-related stroke and stroke-prone status. Details of the stroke-prone status and preventions inflicted at this stage remain to be explored.

We hope that aspects reflected in this review will inspire improved stroke awareness and prevention in countries facing an upward trend and high rates of the disease, similar to China. The odd ratios of hypertension, atrial fibrillation and alcohol intake for stroke in Southeast Asia and Africa are consistently higher than those in western countries [16]. The prevalence of smoking and drinking as well as the tobacco and alcohol consumption are at an overwhelmingly high level in Southeast Asian countries, particularly in Indonesia and Philippines where the policy of tobacco and alcohol is as weak as that in China [1, 48, 96]. With impact of extreme cold weathers due to Siberian cold current and declines of Arctic sea-ice, Mongolia that has the largest increase of stroke incidence worldwide and Russia that has completely high stroke incidence need to note the impact of cold waves to stroke as well [1, 97].

Collectively, aspects discussed above generated from the outlier (China) with increasing incidence of stroke are confronted with great challenges, but they are potential, modifiable and promising. Vigorous interventions and further research are expected from collaboration and efforts of different parts since primary prevention of stroke is the most cost-effective way to reduce the health burden.
